# Spatial relationship between telocytes, interstitial cells of Cajal and the enteric nervous system in the human ileum and colon

**DOI:** 10.1111/jcmm.15013

**Published:** 2020-01-26

**Authors:** Béla Veress, Bodil Ohlsson

**Affiliations:** ^1^ Department of Pathology Skåne University Hospital Malmö Sweden; ^2^ Department of Internal Medicine Skane University Hospital Lund University Malmö Sweden

**Keywords:** enteric nervous system, human bowel, immunohistochemistry, interstitial cells of Cajal, telocytes

## Abstract

Telocytes (TCs) are recently described interstitial cells, present in almost all human organs. Among many other functions, TCs regulate gastrointestinal motility together with the interstitial cells of Cajal (ICCs). TCs and ICCs have close localization in the human myenteric plexus; however, the exact spatial relationship cannot be clearly examined by previously applied double immunofluorescence/confocal microscopy. Data on TCs and submucosal ganglia and their relationship to intestinal nerves are scarce. The aim of the study was to analyse the spatial relationship among these components in the normal human ileum and colon with double CD34/CD117 and CD34/S100 immunohistochemistry and high‐resolution light microscopy. TCs were found to almost completely encompass both myenteric and submucosal ganglia in ileum and colon. An incomplete monolayer of ICCs was localized between the TCs and the longitudinal muscle cells in ileum, whereas only scattered ICCs were present on both surfaces of the colonic myenteric ganglia. TC‐telopodes were observed within colonic myenteric ganglia. TCs, but no ICCs, were present within and around the interganglionic nerve fascicles, submucosal nerves and mesenterial nerves, but were only observed along small nerves intramuscularly. These anatomic differences probably reflect the various roles of TCs and ICCs in the bowel function.

## 1 INTRODUCTION

It is widely accepted that the gastrointestinal peristaltic movement is under the influence of/governed by the myenteric plexus and the interstitial cells of Cajal (ICCs). The other component of the enteric nervous system (ENS), the submucosal plexus, does not take part in the regulation of the bowel movement, but influences the secretory activity apart from that of the endocrine cells, mucosa and blood vessels.^1^ The ICCs are organized into several locations, with variation between different gastrointestinal regions. In both the small and large intestine, ICCs have been found around the myenteric ICC plexus (ICC‐MP), within the circular and longitudinal muscle layers (ICC‐IM) and in the interlamellar connective tissue of the circular muscle.^2^, ^3^, ^4^, ^5^, ^6^, ^7^, ^8^, ^9^ Additionally, ICCs form/build up the deep muscular ICC plexus between the inner and outer circular muscle (ICC‐DMP) in ileum,^10^, ^11^, ^12^ and the submuscular plexus (ICC‐SMP) at the submucosal‐circular muscle border in colon.^11^, ^12^, ^13^

Immunohistochemical studies have since the 1990s revealed another fibroblast‐ or ICC‐like cell in similar localization as ICCs, but with different phenotype: negative for *c‐kit* (CD117) and positive for CD34 and platelet‐derived growth factor receptor α (PDGFRα).^12^, ^14^, ^15^ The term telocyte (TC) was coined in 2010 by Popescu and Faussone‐Pellegrini for these ICC‐like cells, and their long and very thin cytoplasmic projections were called telopodes.^16^ TCs have been localized in several other organs as well.^17^ Several investigations have suggested that TCs and PDGFRα‐positive cells influence the intestinal motility, among many other functions.^12^, ^18^, ^19^, ^20^, ^21^

Electron microscopic studies showed that both ICCs and fibroblast‐like cells were present around the myenteric ganglia of the human small and large intestine.^5^, ^9^ Double immunofluorescence, combined with confocal microscopy, proved that CD34‐immunoreactive, fibroblast‐like cells were distinct but closely attached to ICCs both in human and mouse stomach, small intestine and colon.^14^, ^22^ These CD34‐positive cells were also S100‐negative and accompanied S100‐positive glial cells and nerve fibres.^14^

The exact spatial relationship between the different cellular components in the myenteric plexus is, however, better analysed in conventional microscope after double immunostainings. The few studies performed regarding ICCs and TC and their relation to the submucosal ganglia have shown varying results.^6^, ^8^, ^11^, ^12^ Therefore, the aim of the present study was to examine double immunohistochemistry for ICCs, TCs and glial/Schwann cells in the light microscope, in regard to the connections between these cells and the neural plexi of the ENS and the small nerves in the muscle layers of human ileum and colon.

## 2 MATERIAL AND METHODS

The study was performed according to the Declaration of Helsinki and approved by the Ethical Review Board at Lund University (2012/527, date of approval 25/10/2012). Since the samples were unidentified samples used as controls, with only sex and age as known variables, the subjects did not have to leave informed consent, according to Swedish ethical rules. The study is not a part of a clinical trial.

Four specimens of normal human ileum and two specimens of normal colon were used for the analyses. The samples were taken from macroscopically normal ileum of right‐sided hemicolectomy resections due to carcinoma (3 men of 55, 60 and 84 years of age, respectively, and one woman, 60 years old) and normal sigmoid colon also resected due to carcinoma (2 men, 65 and 71 years old). The specimens were fixed in buffered formalin overnight and embedded in paraffin. Serial sections of 4 μm thickness were deparaffinized and stained by haematoxylin & eosin. For immunohistochemistry, CC1 ph 8.5 buffer (Ventana Medical Systems) was applied for the deparaffinized unstained sections before applying the antibodies. The following primary antibodies were used: rabbit monoclonal antiserum to human *c‐Kit* (CD117; dilution: 1:500; clone: YR145; Epitomic, Abscam Co), mouse monoclonal antibody against to human CD34 (RTU prediluted; clone QBEND/10; catalogue no. 790‐2927; Ventana Medical Systems) and polyclonal antibody to bovine brain S100 (dilution: 1:800; catalogue: Z0311; Agilent Technologies). For detection of the immunostaining, Ultra View DAB Kit (brown colour) and Ultra View Fast Red Kit (red colour) (both Ventana Medical Systems) were used in Ventana's BenchMark ULTRA automated immunostainer system.

With double immunohistochemistry, either CD117 was detected first with Fast Red Kit followed by CD34 with DAB Kit, or CD34 was detected first with DAB Kit followed by S100 with Fast Red Kit. The double stainings were also performed in reverse order. As internal control served endothelium of blood vessels (CD34^+^) and mast cells (CD117). As negative controls, the primary antibodies were omitted and replaced by serum. Nuclear counterstain was Mayer's haematoxylin.

Quantification of TCs and ICCs was performed in the myenteric plexus and in the circular muscle layer. Only immunoreactive cells with clearly seen cellular nuclei were counted. In the myenteric plexus, 1 mm long segments were counted at 5 different places, and in the circular muscle, 5 of the most cellular areas (high power field: HPF; ×40, 0.65 objective lens, ×10 ocular) were chosen. The results were given as mean of all individual counting.

## 3 RESULTS

### 3.1 Myenteric plexus

#### 3.1.1 Ileum

**Figure 1:**
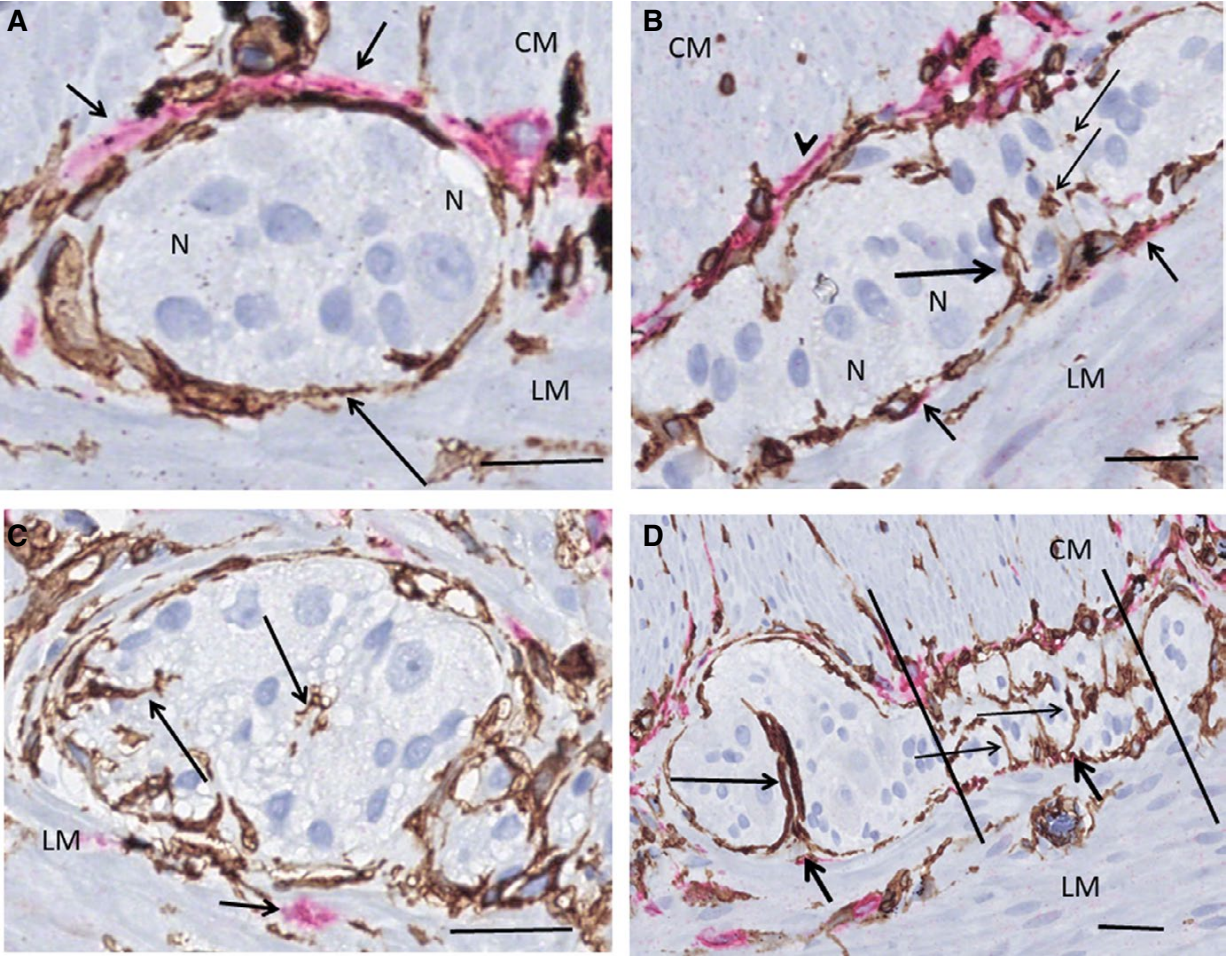
Ileum. Myenteric ganglia. Double labelling, CD34/c‐Kit; telocytes (TC): brown, interstitial cells of Cajal (ICC): red. A, TCs are in close contact with the ganglion forming a continuous layer (long arrow). The ICCs are situated between the TCs and the circular muscle (short arrows). No ICCs are observed towards the longitudinal muscle and no telopodes within the ganglion. B, Double layer of TCs and ICCs towards the circular muscle (arrowhead). Projections of two ICCs towards the longitudinal muscle (short arrows) and a telopode entering the ganglion (long arrow), probably near the origin of the interganglionic fascicle. A few transversally sectioned telopodes are also observed (thin arrows). C, Probable terminal part of a ganglion with telopodes (long arrows). There is ICC present outside the TC layer (short arrow). D, Terminal part of a ganglion with the beginning of the interganglionic nerve bundle (between the lines). There are telopodes of TCs (thin arrows) and only nuclei of glial cells are within this area. A telopode forms a semi‐septum within the ganglion (long arrow). Note a few scattered ICC projections among TCs towards the longitudinal muscle layer (short arrows). CM, circular muscle layer; LM, longitudinal muscle layer; N, neuron. Scale bars: 25 µm on actual figures

**Figure 2:**
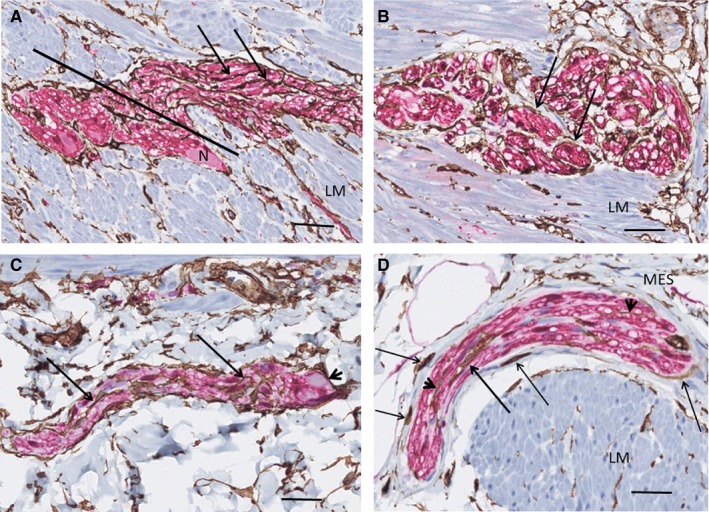
Ileum. Nerve fascicles. Double labelling, CD34/S100; telocytes (TC): brown, Schwann/glial cells: red. A, Terminal part of myenteric ganglion with interganglionic nerve fascicle to the right of the line. TCs/telopodes (arrows) run parallel with the Schwann cells. B, A myenteric nerve fascicle partly transversally cut. The TCs/telopodes (arrows) encompass small groups of axons. C, Submucosal nerve. The telopodes (arrows) are parallel with axons. On the right, the terminal part of a ganglion with a neuron (arrowhead). D, Mesenterial nerve with intraneuronal TC (thick arrow) and telopodes (arrowheads). Extraneural TCs partly encompass the nerve (thin arrows) with or without thin collagen fibres in between. Note the almost complete layer of TCs at the muscular‐submucosal border. G, ganglion; LM, longitudinal layer; MES, mesenterium; N, neuron. Scale bars: 25 µm on actual figures

The TCs and ICCs of ICC‐MP formed either an almost complete double layer at the inner surface of the ganglia, facing the circular muscle with the TCs always in between the ganglia and the ICCs, or there was only one very thin layer of TCs or telopodes, with or without a few ICCs/projections intermingling with TCs. On the outer surface of the ganglia towards the longitudinal muscle layer, only a few ICCs and their small projections were scattered partly intermingled with ICC, without the formation of a double layer (Figure 1A‐D). Quantification of TCs and ICCs in the myenteric plexus showed 12 TCs per mm vs 24 ICCs per mm. Few telopodes were observed within the ganglia, probably at the beginning of interganglionic nerve fascicles, in contrast to ICC projections, which were never seen within the ganglia (Figure 1B, 1). The myenteric nerve fascicles and nerve fibres in the submucosa and mesenterium were ‘covered’ by TCs, and within these nervous structures, TC and telopodes were always present parallel with axons, dividing the nerve bundles into smaller groups of axons (Figure 2A‐D). ICCs were scattered at the outside of interganglionic fascicles, but no ICC projections were observed within the nerves (data not shown).

#### 3.1.2 Colon

**Figure 3:**
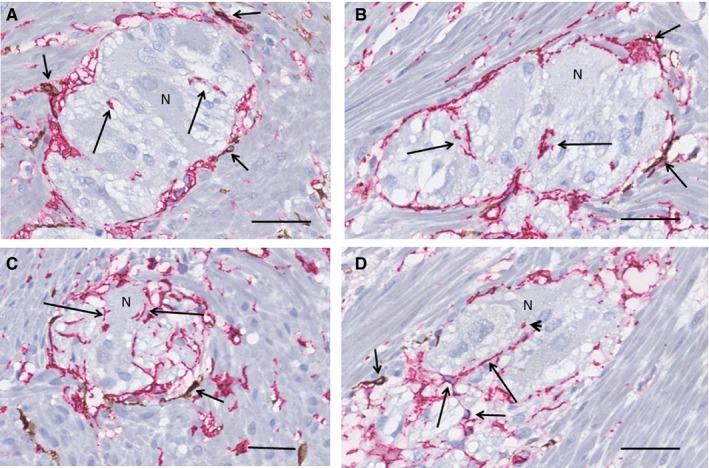
Colon. Myenteric ganglia, surrounded by longitudinal muscle cells (transversal section). Double labelling, c‐Kit/CD34; telocytes (TC): red, interstitial cells of Cajal (ICC): brown. A, An almost complete layer of TCs surrounds the ganglion with only a few scattered ICCs (short arrows). Short segments of telopodes are within the ganglion (long arrows), some are at close contact with neuron. B, The location of the few ICCs around the ganglion (short arrows) is similar to that of the ileum, that is between the telopodes and the smooth muscle cells. There are telopodes within the ganglion (long arrows). C, Telopodes are in close contact with neurons along their plasma membrane (long arrows). Short arrow shows ICCs. D, Several telopodes (long arrows) are within the ganglion even with contact with one of the neurons (arrowhead). Short arrows point to ICCs. N, neuron. Scale bars: 25 µm on actual figures

The pattern of TCs and the localization of ICCs around the ganglia were similar in the colon to those of the ileum with two exceptions: (a) the number of ICCs was substantially less with only a few ICCs scattered around the ganglia and (b) telopodes were usually observed within the ganglia in close localization to neurons (Figure 3A‐D). The quantification of TCs and ICCs in the myenteric plexus showed 35 TCs per mm vs 5 ICCs per mm.

The myenteric nerve fascicles, submucosal nerves and mesocolic nerves showed similar arrangements of TCs/telopodes as in the ileum. ICCs were not present within the interganglionic fascicles (data not shown).

### 3.2 Submucosal ganglia in the ileum and colon

**Figure 4:**
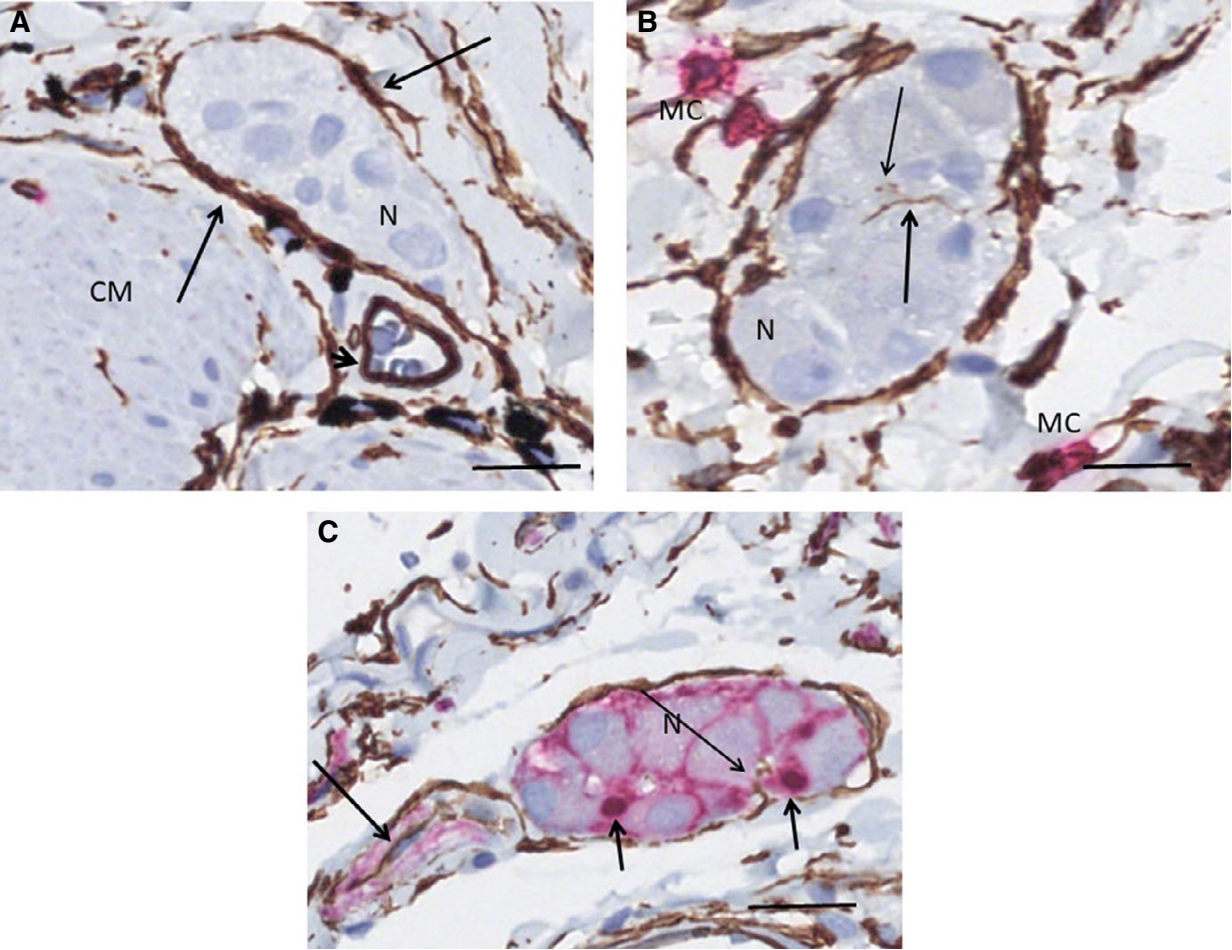
Submucosal ganglia. A, B ileum. Double labelling, CD34/c‐Kit, telocytes (TC): brown, interstitial cells of Cajal (ICC)/mast cells: red; C colon, double labelling, CD34/S100, TC: brown, Schwann cells: red. A, The ganglion is encompassed by a layer of TCs (long arrows). No ICCs are around the ganglion. Arrowhead shows blood vessel with CD34^+^ endothelial cells and red blood cells in the lumen. B, A long telopode (thick arrow) and a few transversally cut telopode (thin arrow) within the ganglion. No ICCs are present. C, TC within a ganglion with a nerve (long arrow). TCs form also an incomplete layer along the nerve. One telopode within the ganglion (thin arrow). The nuclei of the glial cells within the ganglion are S100+ (short arrows). CM, circular muscle layer; MC, mast cells; N, neuron. Scale bars: 25 µm on actual figures

Telocytes were present in a single ‘TC‐sheath’ encompassing the submucosal ganglia in both the ileum and colon, but ICCs never occurred in this localization (Figure 4A‐C). Occasionally, telopodes entered the ganglion (Figure 4B, 4). Within the nerves, the TCs/telopodes showed a pattern similar to the myenteric fascicles, as well as to the nerves within the mesenterium (Figure 4C).

### 3.3 Small nerve fibres in the muscle layers

**Figure 5:**
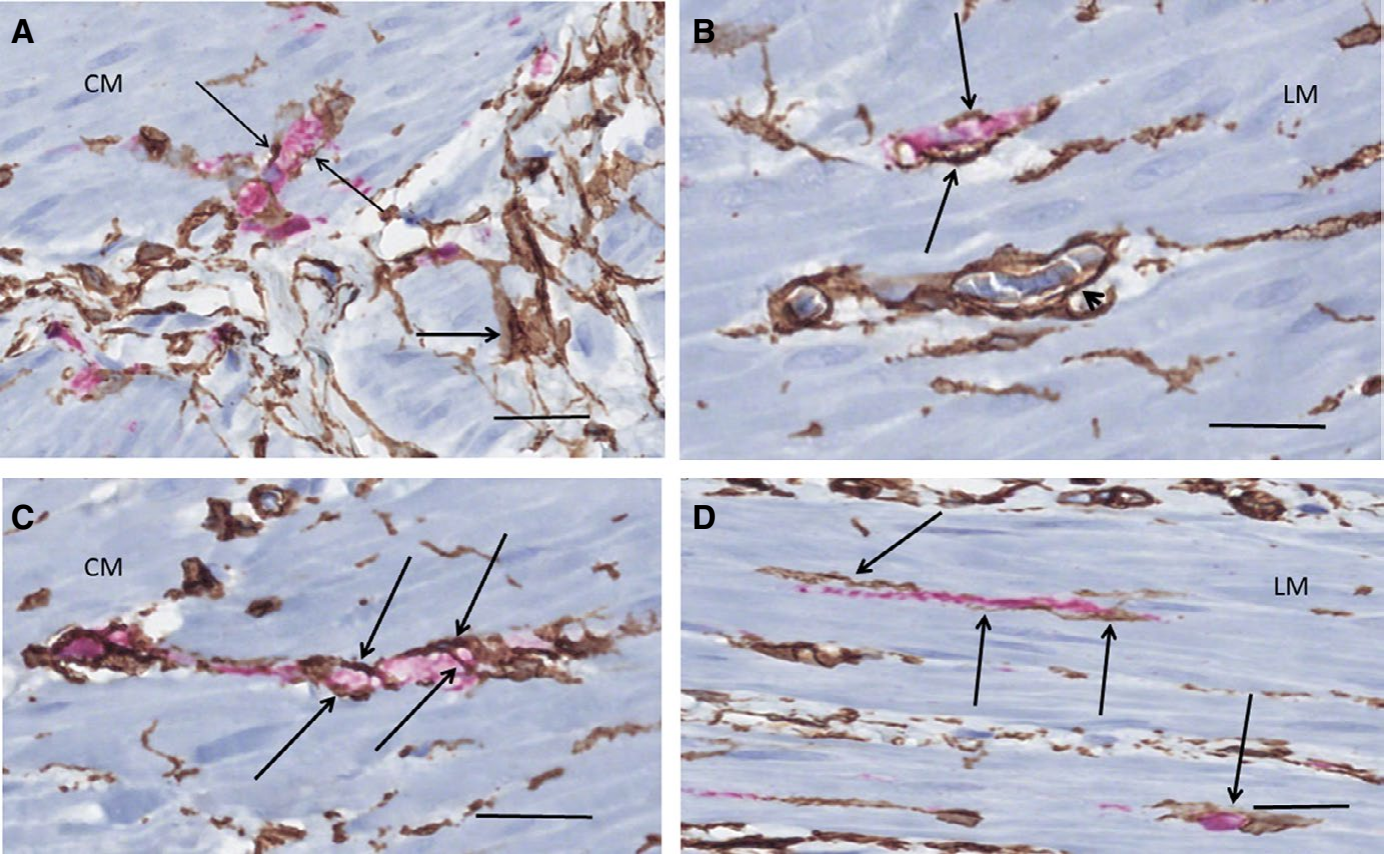
Small nerves within the muscle layers. A, B, D ileum, C colon. Double labelling, CD34/S100, telocytes (TC): brown, Schwann cells: red. A, TCs are on both sides of the 8 μm thick nerve (thin arrows). Several tangentially sectioned TCs with long telopodes forming a network (thick arrow shows one of the TCs). B, 8 μm thick nerve with TC/telopode (arrows) along the nerve. Arrowhead shows CD34 + endothelial cells of a small blood vessel with red blood cells in the lumen. C, 9 μm thick nerve in the middle with telopodes crossing from one side to the other (arrows). D, Nerve fibre thinner than 5 μm with accompanying telopodes (arrows). CM, circular muscle; LM, longitudinal muscle. Scale bars: 25 μm on actual figures

On transversal section within the muscles, TCs formed a network with long telopodes (Figure 5A, right lower corner). TCs followed the small nerves in both ileum and colon, but intraneural TCs could not be observed within the muscle layers. Nerves larger than 5 μm thick were surrounded by a ‘network’ of TCs (Figure 5A‐C), whereas TCs/telopodes were present in a scattered way along the smaller nerves (Figure 5D).

### 3.4 TCs and ICCs between the muscle layers, intramuscularly and in the interlamellar space

**Figure 6:**
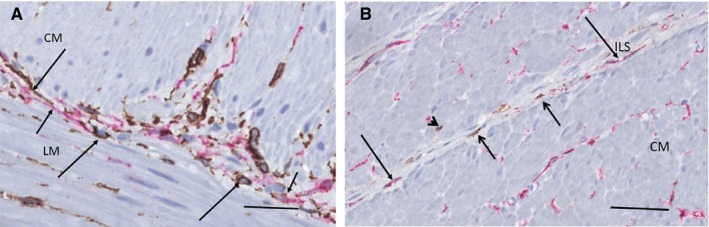
Connective tissue layer between the muscle layers in ileum (A) and interlamellar septum of the circular muscle in colon (B). A, Double labelling, CD34/c‐Kit, telocytes (TC): brown, interstitial cells of Cajal (ICC): red. Within the intermesenteric connective tissue layer, TCs/telopodes (long arrows) and ICCs/projections (short arrows) are scattered. B, Double labelling, c‐Kit/CD34, telocytes (TC): red, interstitial cells of Cajal (ICC): brown. Few projections of ICCs (short arrows) and telopodes of TCs (long arrows) are within the thin interlamellar septum. Several TCs are scattered within the muscle but only one ICC projection (arrowhead). CM, circular muscle layer; ILS, interlamellar septum; LM, longitudinal muscle. Scale bars: 25 μm on actual figures

TCs and ICCs of the ICC‐IM were found in both muscle layers; similar observation was made in the interlamellar septa of the circular muscle (Figure 6 A‐B). Both TCs and ICCs were scattered diffusely in the intermuscular connective tissue layer (Figure 6A) and within the narrow interlamellar space between the muscle lamella of the circular muscle (Figure 6B).

The quantification of TCs and ICCs in the circular muscle layer showed 8 TCs per HPF in ileum and 6 TCs per HPF in colon, respectively, compared to 2 ICCs per HPF in both organs.

## 4 DISCUSSION

This study showed that TCs form an encompassing almost complete sheath around both myenteric and submucosal ganglia and interganglionic fascicles in both ileum and colon. Their telopodes entered the colonic myenteric ganglia. TCs with long telopodes were present along and within all the larger nerve fascicles and peripheral nerves of the bowel and the mesenterium. In contrast, ICCs formed a layer outside the TC layer of the myenteric ganglia in the ileum and were scattered in similar localization at the interganglionic fascicles. In the colon, ICCs were scarce and only scattered on both surfaces of the myenteric ganglia intermingled with TCs. These observations are in agreement with and support previous findings obtained in confocal microscopic analysis,^14^, ^22^ but are to some part novel data.

In the ileum, TCs and ICCs were either localized in parallel in two complete separate layers on *the inner surface* of the myenteric ganglia with TCs between the ganglia and the ICCs, or with TC and ICCs intermingling with each other, corroborating with previous observations by Vannucchi et al^23^ and Milia et al.^24^ However, on *the outer surface* of the ileal ganglia and of the colonic ganglia, there was only one almost complete TC layer with scattered ICCs, in agreement with previous reports.^6^, ^9^, ^23^, ^25^ TCs were not found within the myenteric ganglia in the ileum, although telopodes appeared within or near the origin of interganglionic fascicles. The intraganglionic pattern of TCs was, however, completely different in the colon with telopodes within the ganglion showing numerous close contacts with neurons, as described by some,^23^ but contradictory to others' description that fibroblast‐like cells never penetrated the ganglia.^9^ ICCs could not be found within the myenteric ganglia in the present and some former studies, 8, 9 whereas some authors have described ICCs and their processes within ganglia in both ileum and colon.^23^, ^24^, ^25^

Telocytes entered the myenteric ganglia at the beginning of the interganglionic nerve fascicles and ran on the surface of the fascicles in both ileum and colon, which has already been demonstrated.^22^, ^23^ The novel findings were that TCs/telopodes were observed within the fascicles and that small intramuscular nerve fibres were surrounded or followed by TCs/telopodes on their surface.

The almost complete TC layer around submucosal plexi confirmed the results from Milia et al^24^ and Manetti et al.^26^ However, no ICCs were observed in this localization, partly in line with previous reports of few ICC in the actual area.^6^, ^8^ Nevertheless, several other studies have described the presence of ICC in close relation to or around the colonic submucosal plexus.^11^, ^12^, ^22^, ^25^

Our findings of TCs and ICCs in the interlamellar septa of the circular muscle layer are well‐known data.^5^, ^8^, ^23^, ^24^, ^27^, ^28^ The numbers of TCs and ICCs can be related to another report which found comparable findings, without given exact localization within the bowel wall.^14^ We have to emphasize that the number of samples in our analysis is too low for any certain conclusion regarding the numerical relation between these two cell types.

Thus, the architecture of the two cell types differs markedly, which may reflect the functional role of these cells. Furthermore, the shape and distribution of TC and their location may vary in different wall layers of ileum, as previously described in a large study cohort.^23^ Whereas the myenteric ganglia in both small and large intestine are organizing the movement of the bowel, the submucosal ganglia take part in the control of the mucosal secretory activities.^1^ ICCs are of great importance for bowel movements, but are not involved in the mucosal functions. On the other hand, TCs probably have a protective role for the various tissue components, and their appearance is more like a monolayer, surrounding the different tissue components, described both in the present and previous studies.^23^ The difference between the ileum and colon regarding the pattern and number of ICCs and TCs, around and within the ganglia, could possibly be explained by the difference in motility between the small and large bowel.^29^ The TCs have an important role in the organization and control of the extracellular matrix, structural support, intercellular communication and creation of the environment.^21^ Furthermore, TCs contribute to the regulation of the Wnt pathway in regeneration and through toll‐like receptors in immunoregulation.^19^, ^30^ Accordingly, patients with Crohn's disease and ulcerative colitis showed a reduced amount of TCs in affected bowel segments, as well as reduced amounts of ICCs.^24^, ^26^ Although the loss of TC and ICC was parallel in inflammatory bowel diseases,^24^, ^26^ findings in diabetes mellitus showed that FLC, proven to be identical to TC,^23^ were more resistant to degeneration than ICC.^31^, ^32^ Hypothetically, these data might explain the histopathological experience that submucosal ganglia are more preserved than myenteric ganglia in dysmotility disorders (personal experience).

Differences between studies may be explained by small sample sizes of most studies. Differences in affinity and specificity of antibodies from different producers and batches may be another explanation to contradictory results. One hypothesis is that malignant diseases may affect tissues at a distance from the local tumour,^33^ although the resection margin looks healthy. Thus, one cannot exclude some loss of ICC in samples from patients obtained during surgery for a malignant tumour.

In conclusion, regarding the localization and number of TCs and ICCs, the difference between human myenteric and submucosal ganglia, nerves and muscle layers on the one hand, and between ileum and colon on the other hand might probably reflect the role of these cells in the function of the bowel, that is the maintenance of homeostasis in the microenvironment and the motility of the bowel, respectively. Further investigations are necessary to prove or disprove this hypothesis.

## CONFLICT OF INTEREST

The authors confirm that there are no conflicts of interest.

## AUTHOR CONTRIBUTIONS

Béla Veress designed and performed the analyses and wrote the main part of the paper. Both authors contributed to the intellectual analysis of the result and approved the final version of the manuscript.

## Data Availability

All data included in this study are available upon request by contact with the corresponding author.
